# Rapid evolution of SARS-CoV-2 challenges human defenses

**DOI:** 10.1038/s41598-022-10097-z

**Published:** 2022-04-19

**Authors:** Carlos M. Duarte, David I. Ketcheson, Víctor M. Eguíluz, Susana Agustí, Juan Fernández-Gracia, Tahira Jamil, Elisa Laiolo, Takashi Gojobori, Intikhab Alam

**Affiliations:** 1 Red Sea Research Centre (RSRC), King Abdullah University of Science and Technology, Thuwal, 23955 Saudi Arabia; 2 Computational Bioscience Research Centre (CBRC), King Abdullah University of Science and Technology, Thuwal, 23955 Saudi Arabia; 3 Computer, Electrical, and Mathematical Sciences and Engineering (CEMSE) Division, King Abdullah University of Science and Technology, Thuwal, 23955 Saudi Arabia; 4 Instituto de Física Interdisciplinar y Sistemas Complejos IFISC (UIB-CSIC), Palma de Mallorca, Spain

**Keywords:** Evolution, Evolutionary theory, Viral infection

## Abstract

The race between pathogens and their hosts is a major evolutionary driver, where both reshuffle their genomes to overcome and reorganize the defenses for infection, respectively. Evolutionary theory helps formulate predictions on the future evolutionary dynamics of SARS-CoV-2, which can be monitored through unprecedented real-time tracking of SARS-CoV-2 population genomics at the global scale. Here we quantify the accelerating evolution of SARS-CoV-2 by tracking the SARS-CoV-2 mutation globally, with a focus on the Receptor Binding Domain (RBD) of the spike protein determining infection success. We estimate that the > 820 million people that had been infected by October 5, 2021, produced up to 10^21^ copies of the virus, with 12 new effective RBD variants appearing, on average, daily. Doubling of the number of RBD variants every 89 days, followed by selection of the most infective variants challenges our defenses and calls for a shift to anticipatory, rather than reactive tactics involving collaborative global sequencing and vaccination.

## Introduction

Building on early ideas by Haldane^1^, the evolutionary race between hosts and pathogens has been described, in a metaphoric sense, by the Red Queen theory^2^. This metaphor refers to the warning of the Red Queen to Alice, in Lewis Carroll’s book^3^, that in her kingdom “it takes all the running you can do, to keep in the same place. If you want to get somewhere else, you must run at least twice as fast as that!”. The repeated discovery of more infectious variants shows that the SARS-CoV-2 virus is already engaged in this evolutionary race.

Here we provide insights into the current and future evolution of SARS-CoV-2 through a macroscopic consideration of evolutionary ecology theory. We first examine the rise of novel SARS-CoV-2 variants and their relationship to human infections, provide evidence of the rates and paths of selection driving the evolution of SARS-CoV-2 and, building on this evidence, discuss the expected outcomes and the most effective defense tactics. We focus our analysis on mutations at the 194 amino-acid RBD of SARS-CoV-2^4,5^. We do so on the basis of a unique resource, based on raw sequenced genomes from GISAID (www.gisaid.org), identifying mutations and Mutation Fingerprints (MF), available through our in-house platform COVID-19 virus Mutation Tracker^5^ (CovMT; https://www.cbrc.kaust.edu.sa/covmt) We define each set of SARS-CoV-2 genomes that generate the same amino acid sequence in the RBD region of the spike protein^4^ as a unique RBD variant (cf. “Methods”).

## Results

### Virus production and mutation

**Figure 1:**
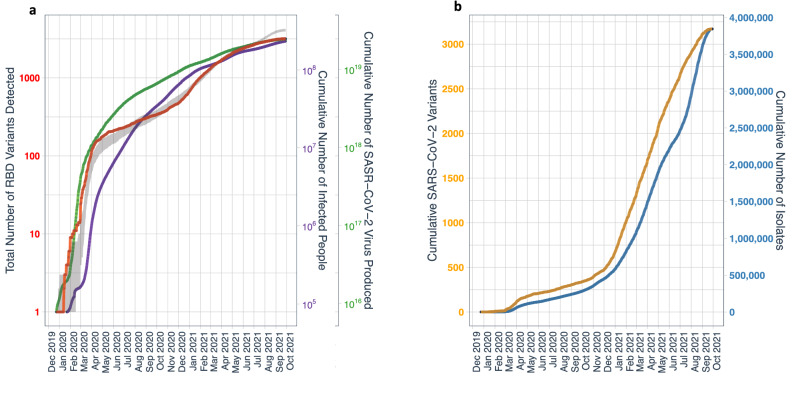
(**a**) Cumulative number of SARS-CoV-2-infected humans (purple line), calculated total number of virus copies produced (green line) until October 5, 2021 (cf. “Methods”), detected (red line) and predicted (grey shaded area, representing minimum and maximum bounds) RBD variants over time. The predicted RBD variants are based on Monte Carlo simulation of viral mutations. New RBD variants are generated based on a mutation rate of 2 per million human infections. The fitness of new variants is taken as a Pareto distribution with scale 10^–6^ and shape parameter ¼. The proportional population of two variants is assumed to change at a rate proportional to the ratio of their fitness, with a characteristic time of 20 days. Isolates are modeled as random samples from the resulting viral population. (**b**) Cumulative number of SARS-CoV-2 isolates sequenced over time (blue line) and cumulative number of detected RBD variants rescaled by a factor of 900 over time (brown line).

The number of copies of the virus produced depends on the number of people infected globally along with the number of copies transcribed per infected subject. The verified number of diagnosed COVID-19 infections is known to drastically underestimate real infections, and the ratio between the two varies greatly depending on time and geographic region^6,7^. Confirmed COVID deaths also underestimate real COVID deaths, but are much more accurate compared to confirmed infections^8,9^. We therefore adopted a model that combines the reported COVID-19 deaths^6^ which is just over 4.8 million worldwide (by October 5, 2021), with an infection fatality ratio to calculate infections^9^. We improved this model to account for the demographics for each country along with age-specific infection fatality ratios^6^. This model estimates the true number of infections by October 5, 2021 at just over 820 million (Fig. 1a). This should be taken as a rough approximation since any estimation of true infections, as well as true COVID-caused mortality, is subject to multiple sources of uncertainties including extreme differences in testing capacity and reporting of covid mortality according to the countries. Assuming a total transcription of about 10^12^ viral genomes per individual along an infection cycle^10^ and provided our cumulative estimate of infected persons, the number of SARS-CoV-2 copies produced globally since the pandemic started is in the range 10^17^–10^21^ by October 5, 2021 (Fig. 1a).

Through July 2021, the growth in the total number of RBD variants closely tracked the calculated cumulative number of virus copies over time, consistent with mutation rate being a function of the number of copies produced (Fig. 1), as every genome transcription is accompanied by a likelihood of mutations. Since then and until October 5, 2021, the number of new variants relative to new isolates has decreased importantly; this may be due to the dominance of the Delta variant, increased sampling or, the most likely explanation, increasing vaccination^11^ leading to reduced infection^12^, viral loads and fewer viral copies produced per infected individual^13–16^. This suggestion should be verified by a dedicated study of how vaccination is affecting realized mutation rates in the global population. The total number of RBD variants reached 3167 by October 5, 2021, yielding 3.85 new variants detected for each million infected persons. Many of the RBD variants present mutations elsewhere in their genome, so that each RBD variant, considering mutations in other parts of the genome, is represented by a number of variants, leading to a total of 1,108,912 unique variants presenting at least one mutation in the RBD region. Many sequenced isolates present mutations in regions of the genome other than the RBD domain, so the total number of SARS-CoV-2 variants (i.e. unique sequences across the entire genome) is much greater (1,471,222 as of October 5, 2021).

**Figure 2:**
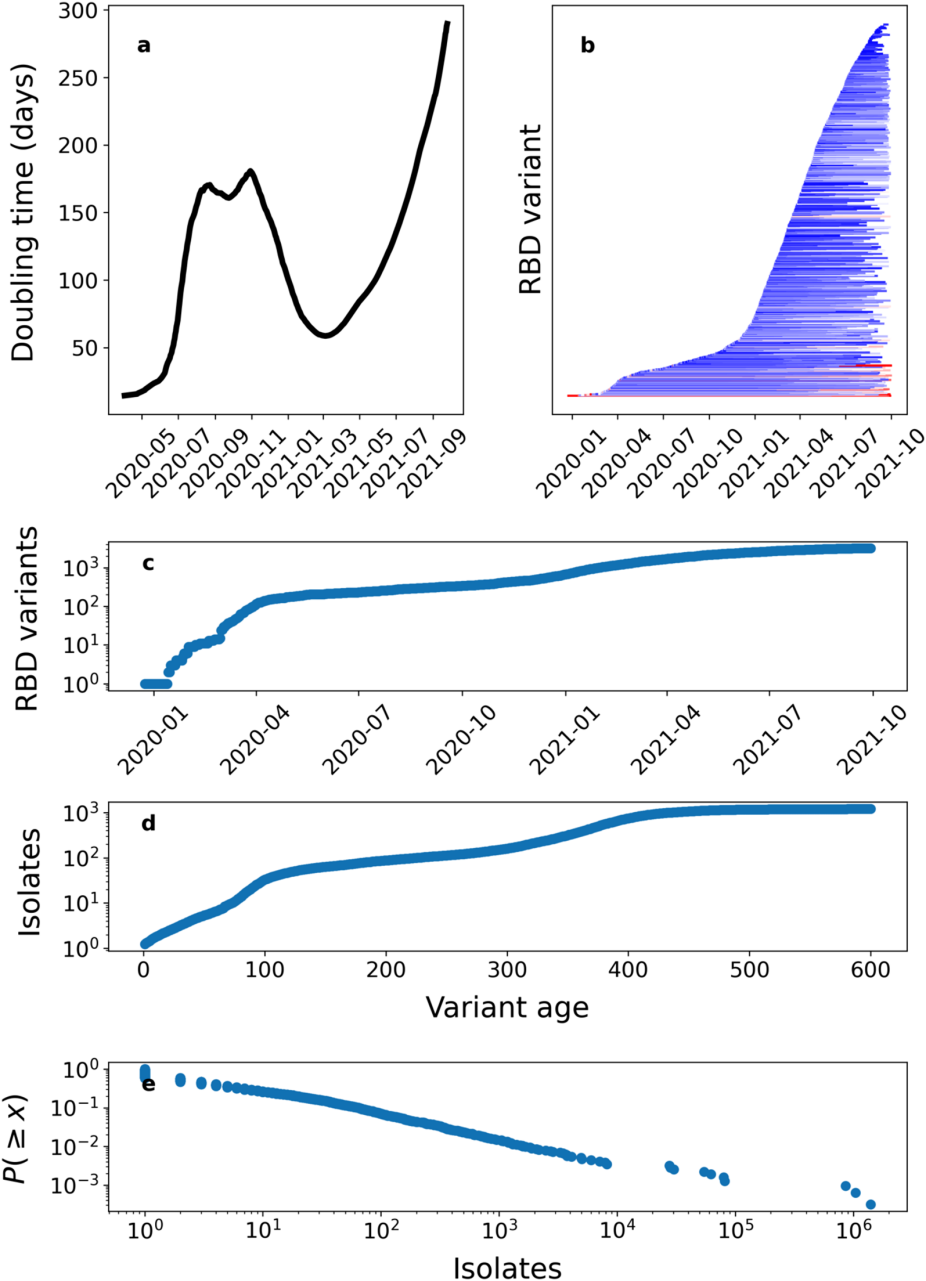
(**a**) Doubling time for RBD variants calculated along a 100 day rolling window. (**b**) Rank abundance distribution of RBD variants, and (**c**) the growth in the number of genome sequences of different unique RBD variants detected from SARS-CoV-2 isolates over time. The figure (**b**) shows a line for each RBD variant along the y-axis that spans from the first day it was observed to the last date it was observed. The color scale denotes the final number of isolates of each RBD variant (increasing from blue to red). Yule’s model of the growth of RBD variants and isolates for each variant, showing the total number of detected unique RBD variants (**c**) and their age (as time since first detection) (**d**) over time, resulting in a power law rank abundance distribution (**e**). Numerically, the characteristic exponent of the exponential growth for RBD variants is 0.00439 ± 0.00004 day^−1^ (SD); then for the average growth it yields 0.00589 ± 0.00004 day^−1^ (SD); leading to an expected exponent of the rank abundance distribution (**e**) of 1.34 (to be compared with 1.45 from the data).

The total number of RBD variants has grown with an overall doubling time of 89 days, but with great oscillations over time (Fig. 1b). The total number of RBD variants grew very rapidly over the first 100 days with a doubling time of 14.4 days (Fig. 2a), indicating that there was already significant diversity in December 2019, consistent with conclusions reported by the WHO mission. The doubling time of the number of RBD variants slowed down to reach a doubling time of 174.6 days 301 days after the first sequenced case, and accelerated again to a doubling time of 58.6 days 437 days after the first genome sequenced. Since, it has slowed down to a doubling time of 289.8 days (Fig. 2a).

The number of unique RBD variants detected is certainly an underestimate of the number of variants in circulation, as their detection depends on sequencing effort, with a total of 3.85 million genome sequences reported for SARS-CoV-2 isolates by October 5, 2021 (Fig. 1b). The rate of detection of new variants strongly depends on sequencing effort, with the ratio between these consistently around 1:900 (Fig. 1b) until July 2021, when this ratio started to increase, which is likely due to reduced production of viral copies per infected person as an outcome of vaccination. Direct modeling of the virus mutation is also capable of reproducing the observed data (Fig. 1b). Our model (cf. “Methods”) suggests that detected RBD variants represent about 60% of all variants, which may be an optimistic estimate since reported isolates represent a biased sample due to testing concentrated in developed nations^17^. The model suggests that about 6 such new variants appear on average for each 1 million human infections. Given that our model estimated 2 million infections daily as of Feb. 15, this implies that 12 new effective variants (i.e. recruited to the virus population) were produced, on average, each day around that period. Reproducing the initial rapid rise in number of detected variants in March 2020 (Fig. 1b) requires a significant level of viral diversity in the initial pool, consistent with recent evidence of a larger-than-reported initial outbreak.

### SARS-CoV-2 evolution and selection

**Figure 3:**
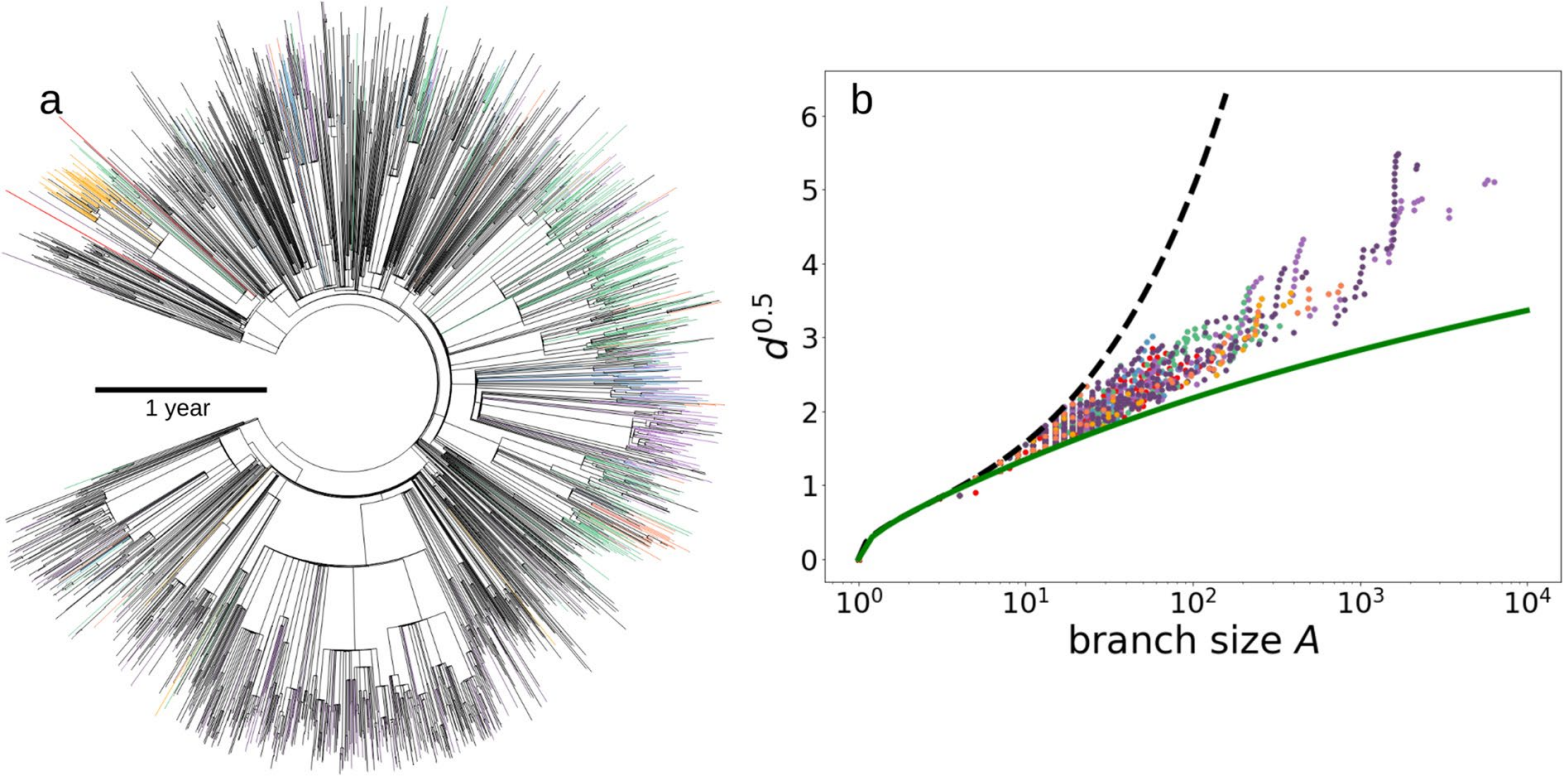
(**a**) Evolutionary tree of the 3167 unique SARS-CoV-2 RBD variants detected by October 5, 2021. The so-called α (B.1.1.7), β (B.1.351), γ (P.1), δ (B.1.617.2), λ (C.37) and μ (B.1.621) lineages are identified as green, blue, purple, deep purple, light orange and dark orange terminal leaves in the tree, respectively. The distance of the branching to the core represents, as indicated by the scale, the time when this branching occurred. (**b**) The relationship between the mean depth (d) and weight of the (A) cluster subtrees in the evolutionary tree. The discontinuous and continuous lines correspond to the two theoretical extreme binary trees: fully asymmetric trees (black dashed line) and fully symmetric (green solid line). The scales of the axes are chosen so that a behavior of the type d ~ (ln A) 2 appears as a straight line. Symbol colors correspond to the so-called α (green), β (blue), γ (purple) and μ (orange) RBD variants.

Evolutionary processes lead to genetic diversification along a branching process, with the evolutionary tree for SARS-CoV-2 RBD variants (Fig. 3a) characterized by a scaling between cumulative branching length and the 1.5 power of subtree size. The cumulative branching length is related to the mean subtree depth, depth = C/A, thus implying that the mean subtree depth scales as the square root of size (Fig. 3a). This scaling is characteristic of protein phylogenies, and deviates from fully balanced (i.e., resulting from lack of selection) and fully imbalanced trees^18^. This evolutionary tree structure is consistent with non-random universal inferred patterns of evolution across scales, from the molecular level [e.g. protein families^19^] to phylogenetic differentiation ranging from micro-evolutionary to macro-evolutionary processes, shaping the diversity of life on the planet^18,19^.

**Figure 4:**
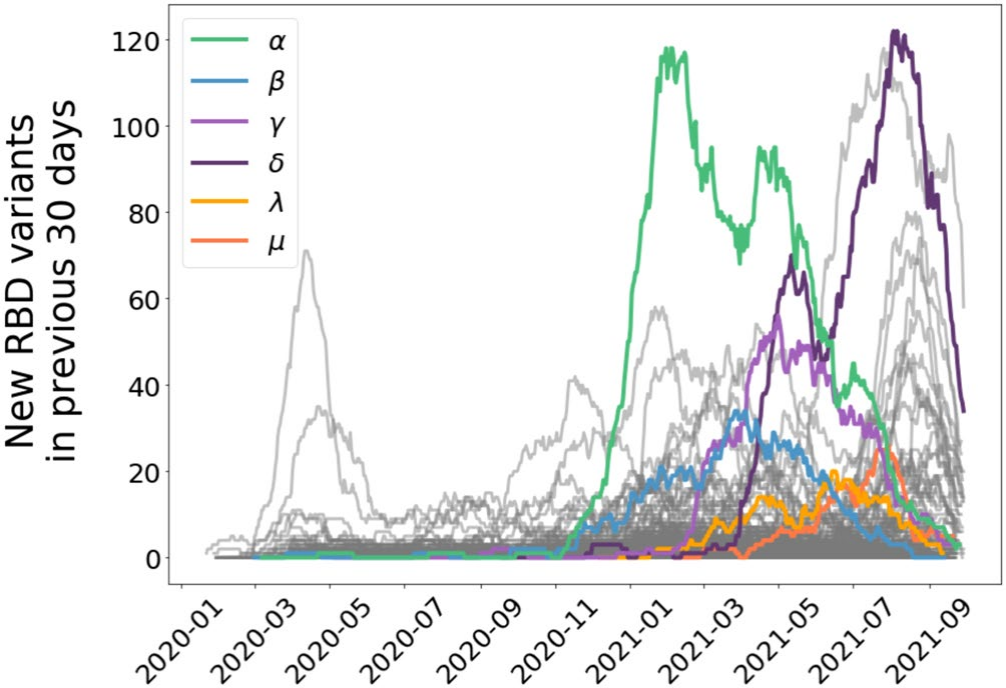
The rate of discovery of new unique RBD variants in the preceding 30 days for each lineage in the SARS-CoV-2 evolutionary tree (Fig. 3a). We identify the variants of interest designated with Greek letters detected through October 5, 2021.

Some RBD variants have rapidly risen to be as represented in the population of sequenced isolates as those identified many months before, with two of the RBD variants exceeding the original variant in number of genotypes sequenced after May 2022 (Figs. 2c and 3a, 4, Video S1). As of Oct 5, 2021, the most represented RBD variants in SARS-CoV-2 genomes are the α and γ variants, with the latter, detected after α, being the most represented, showing a selection for infectivity. These highly successful variants include, specifically, the so-called α variant (B.1.1.7), in which a specific mutated amino acid sequence seems to have appeared independently multiple times (Fig. 3a). Indeed, the α variant has diversified more and faster than other lineages in the RBD region (Fig. 4). As a result, the branch containing most of the α variants has diversified greatly leading to heavy branches in the SARS-CoV-2 RBD evolutionary tree, with one of the branches rapidly diversifying between August and October, 2021 (Fig. 3a, Video S1). The β (B.1.351) and γ (P.1 as well as P.2) RBD variants (Fig. 3) are also being clearly selected in the SARS-CoV-2 RBD evolutionary tree (Fig. 3, Video S11), with the γ variant being the most prevalent one by October 5, 2021. A further indication of rapid evolution and selection is the rapid progression of the development of a hierarchical distribution of the abundance of the various RBD variants (Video S1), to conform to a power law consistent with the Yule law (Fig. 2c–e, Fig. S2), a long-standing empirical observation for large groups of organisms^20^, which requires exponential growth in the number of taxa (here RBD variants) in a lineage (here SARS-CoV-2) followed by exponential growth within each variant, as clearly seen in the evolutionary tree retrieved for SARS-CoV-2 RBD variants (Fig. 3a).

## Discussion

There has been much discussion on the role of impacts on biodiversity in facilitating the arousal of zoonosis^21^. However, the role of the huge, globally-connected human population, in the massive production of viruses propelling the rapid evolution of SARS-CoV-2 has not been sufficiently acknowledged.

Our analysis provides evidence for extraordinarily rapid evolution and selection of SARS-CoV-2, with the number of unique RBD variants doubling every 89 days, which has clearly reached full speed in the Red Queen race, risking outpacing that of human defenses. The same RBD variant, or identical-sequence variant, may arise independently in different locations, as the evolutionary tree suggests for the amino acid sequence shown by the α variant (Fig. 3a). However, the SARS-CoV-2 evolutionary process deviates from a random process, with unbalanced branch development providing evidence of strong selection (Fig. 3a,b), consistent with the dynamics observed for the phylogenesis of protein families^18^. Selection processes remove branches that are not infective while leading to heavy branches of the more infective strains (Fig. 3a). Indeed, the number of copies of the different RBD variants over time is not random, but are under selective pressure, particularly determined by the infectivity of the new variants emerged, as documented for the so-called α (B.1.1.7), β (B.1.351), and γ (P.1 as well as P.2) RBD variants of SARS-CoV-2 (Video S1, Fig. 4). The result of this process is a highly hierarchical dynamic distribution of RBD variants, with a rank-abundance structure conforming to Yule’s law^20^, with just 3 variants (the original one, α and γ) containing 85% of the total isolates (Fig. 2c–e). New, highly infective variants can rapidly recruit to this dominant pool (Video S1). Increased vaccination coverage of efficient vaccines should be able to curve this process by reducing the global production rate of SARS-CoV-2 and, hence, its diversification rate, as the evidence for a change in the relationship between total number of variants and total number of isolates provided here suggests, which deserves deeper and dedicated attention.

High mutation rates of RNA viruses, caused by error-prone RNA-dependent RNA polymerases^22^ along with the huge virus production mediated by the huge pool of available human hosts propel the rapid evolution of SARS-CoV-2. The presence of a large number of variants in circulation within the same host population activates an additional mechanism, recombination, for virus diversification. Recombination involves the formation of chimeric molecules from parental genomes of mixed origin^22^, which likely contributes to the rapid diversification of SARS-CoV-2. Provided a doubling time of SARS-CoV-2 RBD variants of 89 days, the number of SARS-CoV-2 RBD variants will continue to expand. This rapid diversification and selection of RBD variants predicts the selection of more infective variants becoming dominant in a highly hierarchical distribution dynamically conforming to Yule’s law. This heralds a new phase in the pandemic, beyond October 15, 2021, characterized by accelerating evolutionary rates of the virus, which will impose new challenges as new variants of concern, such as the newly detected omicron (B.1.1.529), add to those already detected. However, virus diversification will be slowed down by reduced viral replication derived from growing immunity acquired by the world population through contact with the circulating virus together with increased coverage of efficient vaccines.

Mutation and, possibly, reassortment propel SARS-CoV-2 to be rapidly evolving, implying that human defense tactics need to be reconsidered if we are to overcome the pandemic well before this declines upon reaching the limitation of available hosts. Evolutionary theory posits that hosts develop evolutionary defenses through recombination under sexual reproduction allowing them to modify their genome to anticipate and prevent pathogen attacks^2,23,24^. This requires selection across generations and catastrophic mortality for SARS-CoV-2 morbidity to be selected against. Our defense mechanisms include protections to avoid contact with the virus, and therapies and vaccines once SARS-CoV-2 enters our bodies. External defenses include social distancing, with strict lockdowns proven across many nations to be the most effective defense mechanism, whatever unpopular, to contain the pandemic, along with wearing protections and emerging uses of nanotechnology for virus detection and interception^25,26^. This effort must be complemented with the continuous development of a diverse suite of universal immunizations, such as multivalent nanobodies^27^ and vaccines, eliciting immune defenses that vary and can defend us against a wide range of RBD variants, existing and forthcoming, as new variants that overcome immune defenses produced by previously infected or vaccinated people arise, as demonstrated by our long experience in coping with the drift and shift of the influenza virus^28^. Indeed, recent reports indicate that the convalescent sera and BNT162b2 mRNA vaccine may not be as effective against some of the variants^29^. Yet, our data shows, encouragingly a slow-down of the doubling time of the number of the RBD variants detected along with a progressive reduction in the number of variants detected per infected person after July, 2021. A likely explanation for this shift in tendency, 17 months after the pandemic was declared, is the increase in the number of vaccinated people globally, a suggestion that requires a dedicated analysis, as indicated earlier.

Evolutionary ecology theory helps formulate predictions on the future behavior of SARS-CoV-2. On the other hand, the COVID-19 pandemic provides an unprecedented opportunity to test evolutionary ecology theory, which has been largely inferential in nature. This is important as never before had an evolutionary process been tracked in real time and with such wealth of openly available genomic data at a global scale. The SARS-CoV-2 validates a number of evolutionary theories and laws, such as the evolutionary underpinning of the partially imbalanced architecture of phylogenetic trees across evolutionary scales^18,19^, the diversification process responsible for the long-standing Yule law^20^, and the more targeted framework of the Red Queen theory^2^ predicting the evolutionary tactics of pathogens.

The development of the vaccine in record time, a feat rendered possible by unprecedented collaboration, has been celebrated as the start of the end of the pandemic. Rather, it may be the beginning of a new phase, where the continuous development of novel and diverse and universal vaccines^30^ represents our main defense against the evolving SAS-CoV-2. A universal coronavirus vaccine would ideally protect against existing and future SARS-CoV-2 variants as well as animal-derived coronaviruses that might cause future zoonotic outbreaks and pandemics^30^. This requires sustained global collaboration, and overcoming the challenges derived from the fact that SARS-CoV-2 primarily infect epithelial cells on mucosal surfaces and have limited contact with the systemic immune system, which reduce responses to systemically administered vaccines^30^. In silico analysis of the effectiveness of current vaccines against plausible RBD variants not yet detected, and the design of new effective vaccines against such variants will enable us to overtake SARS-CoV-2 in the evolutionary race, as a reactive, catch-up tactic, as that played to date, will carry continuous risks. Indeed, in silico analysis of detection^31^, infectivity^32^ and vaccine design^33^ of existing and future variants, represent a model for the growing use of in silico prediction as a tool to anticipate defenses for the pandemic. Artificial Intelligence may further help analyze the immunogenicity of all the nonsynonymous variations across described and predicted SARS-CoV-2 sequences to generate a blueprint for effective vaccine development^34^, considering that infectivity is the main driving force of SARS-CoV-2 variant selection. However, increased vaccination and collaborative efforts in SARS-CoV-2 sequencing enabling the early detection of new variants of concern^17^ remain essential strategies to control the pandemic.

## Methods

### COVID-19 cases

The number of positive tests for COVID-19 virus infections reached ~ 100 million in January, 2021; see https://covid19.who.int. To infer real infections over time, we use numbers of confirmed deaths (https://github.com/CSSEGISandData/COVID-19). We apply a regularized deconvolution with an estimated time-to-death distribution to infer the number of real infections over time, as described in Ketcheson et al.^6^.

### COVID-19 virus isolate genomes

Largest resource of Isolate genomes in COVID-19 virus is available at the Global Initiative on Sharing Avian Influenza Data (GISAID, www.gisaid.org)^35^. As of October 5, 2021, more than 3.85 million SARS-CoV-2 genomes are available from around the world.

### COVID-19 virus variants

Mutations in the genome of SARS-CoV-2 are the basis to define its genomic variants. There are several ways to group mutations in COVID-19 virus. GISAID provides generic clades, and more detailed lineages are provided by Phylogenetic Assignment of Named Global Outbreak LINeages (PANGOLIN) tool by Rambaut et al.^36^.

In our effort of a daily updated COVID-19 virus Mutation Tracking system [CovMT, https://www.cbrc.kaust.edu.sa/covmt, Alam et al.^5^], we provide mutation fingerprints (MFs). A mutation fingerprint is defined based on all synonymous and nonsynonymous mutations in an isolate genome. To avoid noise and sequencing errors, a minimum frequency of a mutation from the global population of isolate genomes is kept at 0.001%. We include information about GISAID clades and PANGOLIN lineages for easy exploration of variants. A daily updated table on counts of MFs grouped by sampling dates and location is available at https://www.cbrc.kaust.edu.sa/covmt/data/Variants/World/World_variants_summary.zip.

### RBD variants

The Receptor Binding Domain (RBD) region of Spike protein in SARS-CoV-2 is an important domain region that facilitates the binding of this virus to host cells. Unique RBD variants are defined as those showing exactly the same amino acid sequence for the RBD region of the Spike protein for SARS-CoV-2. We group SARS-CoV-2 genomes into RBD variants by taking the subset of Mutation Fingerprints restricted to the RBD region only and considering only the amino acid mutations. To reduce noise, each individual mutation in the mutation fingerprints is supported with global genome population frequency of at least 0.001%. Taking into account genome sequences in GISAID appear from different sequencing technologies and varying coverages (e.g. long sequence technologies with low coverage) we considered very low frequency mutations as potentially representing noise, since complete information on sequencing coverage is not available with all genomes processed. Mutation Fingerprints of RBD variants as well as all other variants with associated metadata are available at CovMT webpage, https://www.cbrc.kaust.edu.sa/covmt/index.php?p=world-variants.

### Mutation modeling

We estimate the number of effective mutations per human infection based on a direct modeling approach using Monte Carlo simulation. New RBD variants are generated based on an assumed mutation rate (chosen to fit the data). The fitness of new variants is taken as a Pareto distribution with scale 10^–6^ and shape parameter ¼. The proportional population of two variants is assumed to change at a rate proportional to the ratio of their fitnesses, with a characteristic time of twenty days. Isolates are modeled as random samples from the resulting viral population.

### Phylogenetic tree for loose RBD variants

We use the phylogenetic tree provided by nextstrain (https://nextstrain.org/ncov/global accessed 05/10/2021), which provides the phylogeny of ~ 3.8 × 10^6^ genomes sampled between December 2019 and October 2021. We identified which samples had any mutation in the RBD region and pruned the tree to contain only those. We end up with 3170 different RBD loose variants. We also identified which of these variants belonged to the α (B.1.1.7), β (B.1.351), γ (P.1), δ (B.1.617.2), λ (C.37) and µ (B.1.621) lineages using data provided by COVID-19 virus mutation tracker (https://www.cbrc.kaust.edu.sa/covmt/). For the analysis of the depth scaling of the tree branches we followed the procedure described in refs. (green), β (blue), γ (purple) and μ (orange).

### Fit to power laws

The fits to power laws in Fig. 2 were performed using maximum likelihood^37^.

## Supplementary Information

Supplementary Information.

Supplementary Video S1.
