# Supplementary material for: Rapid evolution of SARS-CoV-2 challenges human defenses
Source: Sci Rep. 2022 Apr 19;12:6457. doi: 10.1038/s41598-022-10097-z (PMC9017738; doi:10.1038/s41598-022-10097-z)
Supplement: Supplementary file 1 — Supplementary Information. [file 41598_2022_10097_MOESM1_ESM.docx]

**Supplementary Information**


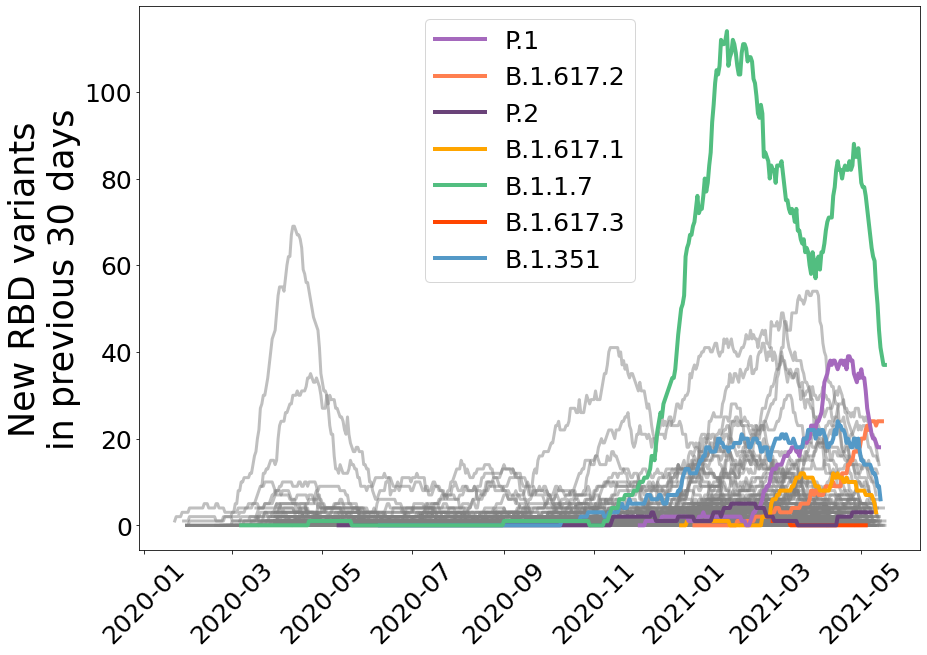


**Supplementary Fig. S1.** The rate of discovery of new unique RBD variants in the preceding 30 days for each lineage in the SARS-CoV-2 evolutionary tree (Fig. 3a). We identify the α (B.1.1.7), β (B.1.351), γ (P.1), δ (B.1.617.2), λ (C.37) and μ (B.1.621) lineages in green, blue, purple, deep purple, light orange and dark orange, respectively. In grey are all the other lineages. While the β, γ, λ and μ do not stand out, the α and δ lineages show a clear excess in diversification in the RBD region as compared with the other lineages, with peaks of around 120 new variants observed in the previous month at the end of January for the α lineage and in august 2021 for the δ lineage.


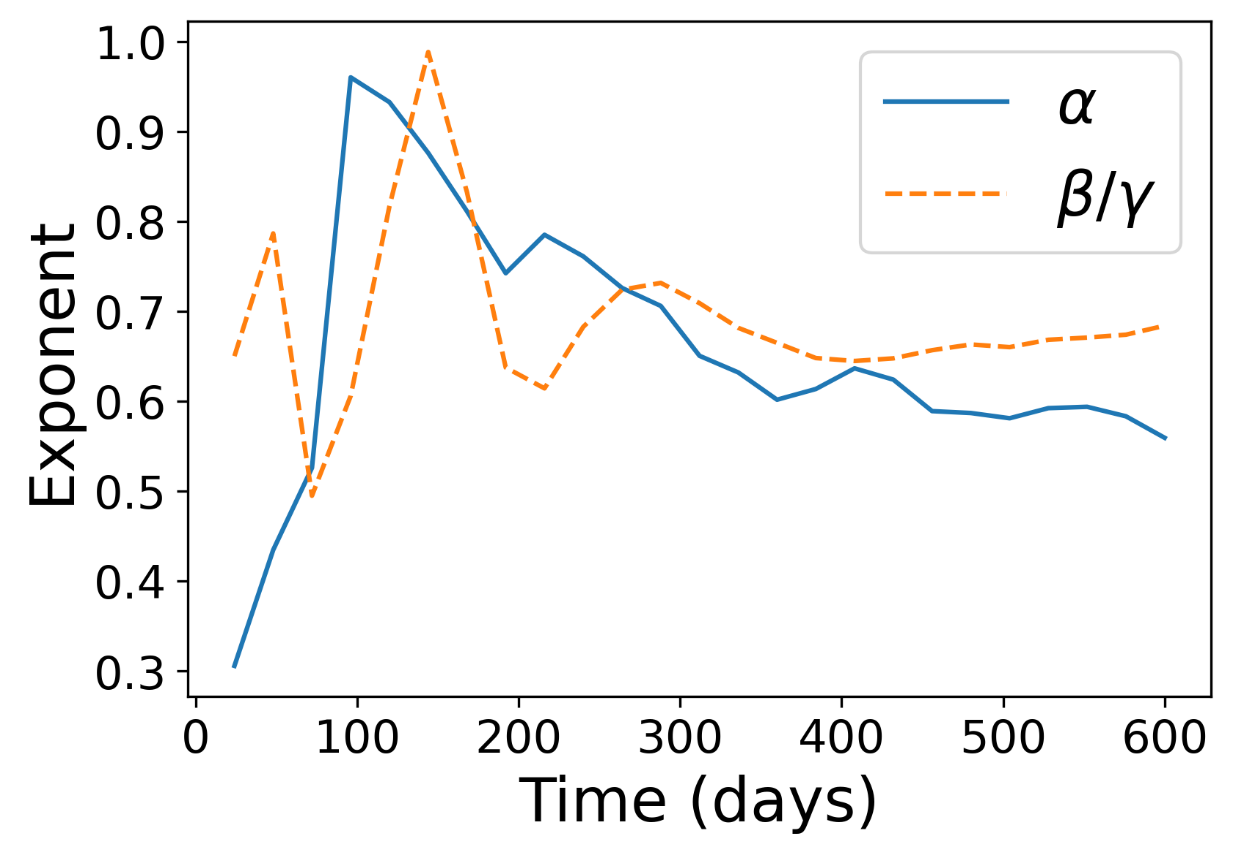


**Supplementary Fig. S2.**Exponent of the power law tail of the variants size distribution (α, blue solid line) and predicted exponent from the Yule model (β/γ, orange dashed line) as a function of the number of days since the first isolate was sequenced. Theory and measurements are in agreement.

Video 1. An animation of the rank distribution of SARS-CoV-2 RBD variants over time, showing the dynamic replacement of new variants among the top 10 variants, in terms of their total representation in the pool of sequenced isolates. As also shown in Figure 2, by mid February 2020, the SARS-CoV-2 population is dominated by the original RBD variant and the RBD:1(N501Y;A23063T) variant. SARS-CoV-2 variants with N501Y mutation were first detected in April, 2020 and these variants re-appeared with more mutations outside the RBD domain during September 2020, and is referred to as the α variant (i.e. B.1.1.7 lineage). The second dominant variant by February 2020 is the RBD:1(S477N;G22992A)​, first identified in March 2020, becoming dominant during summer until December 2020, when the α variant exceeded its abundance. The third variant most found in isolates by mid February 2021 is the S_RBD:1(N439K;C22879A), first detected in March 2020, increasing in number of isolates sequenced after October 2020. Another RBD variant with an increase in the number of SARS-CoV-2 genomes sequenced, just after L452R, is the one with mutation E484K. Variants with E484K mutation got attention when appeared with three RBD mutations RBD:3(K417N;G22813T|E484K;G23012A|N501Y;A23063T) referred to as the β variant (lineage B.1.351). Since, and until October 5, additional variants of concern have been detected, including the variants γ (P.1), δ (B.1.617.2), λ (C.37) and μ (B.1.621). A timeline of top 10 RBD variants can be seen in Figure 4 and at CovMT dedicated page, <https://www.cbrc.kaust.edu.sa/covmt/index.php?p=top-rbd-variants-line> . The video was produced using the package bar-chart-race in python (https://pypi.org/project/bar-chart-race/).
